# pol-miR-731, a teleost miRNA upregulated by megalocytivirus, negatively regulates virus-induced type I interferon response, apoptosis, and cell cycle arrest

**DOI:** 10.1038/srep28354

**Published:** 2016-06-17

**Authors:** Bao-cun Zhang, Ze-jun Zhou, Li Sun

**Affiliations:** 1 Key Laboratory of Experimental Marine Biology, Institute of Oceanology, Chinese Academy of Sciences, Qingdao, China; 2 Laboratory for Marine Biology and Biotechnology, Qingdao National Laboratory for Marine Science and Technology, Qingdao, China; 3 University of Chinese Academy of Sciences, Beijing, China

## Abstract

Megalocytivirus is a DNA virus that is highly infectious in a wide variety of marine and freshwater fish, including Japanese flounder (*Paralichthys olivaceus*), a flatfish that is farmed worldwide. However, the infection mechanism of megalocytivirus remains largely unknown. In this study, we investigated the function of a flounder microRNA, pol-miR-731, in virus-host interaction. We found that pol-miR-731 was induced in expression by megalocytivirus and promoted viral replication at the early infection stage. *In vivo* and *in vitro* studies revealed that pol-miR-731 (i) specifically suppresses the expression of interferon regulatory factor 7 (IRF7) and cellular tumor antigen p53 in a manner that depended on the integrity of the pol-miR-731 complementary sequences in the 3′ untranslated regions of IRF7 and p53, (ii) disrupts megalocytivirus-induced Type I interferon response through IRF7, (iii) inhibits megalocytivirus-induced splenocyte apoptosis and cell cycle arrest through p53. Furthermore, overexpression of IRF7 and p53 abolished both the inhibitory effects of pol-miR-731 on these biological processes and its stimulatory effect on viral replication. These results disclosed a novel evasion mechanism of megalocytivirus mediated by a host miRNA. This study also provides the first evidence that a virus-induced host miRNA can facilitate viral infection by simultaneously suppressing several antiviral pathways.

Iridoviruses are double-stranded DNA viruses that are severe pathogens of fish and amphibians^1^. The family *Iridoviridae* comprises of five genera: *Iridovirus*, *Chloriridovirus*, *Ranavirus*, *Lymphocystisvirus*, and *Megalocytivirus*^2^. *Megalocytivirus* is the causative agent of high-mortality diseases in a wide arrange of marine and freshwater fish^3^. Based on phylogenetic analyses of the genes encoding the major capsid protein and ATPase, the genus *Megalocytivirus* can be divided into three clusters represented by red sea bream iridovirus (RSIV), infectious spleen and kidney necrosis virus (ISKNV), and turbot reddish body iridovirus (TRBIV)^3^. To date, many studies have shown that iridoviruses, mainly *Iridovirus*, *Chloriridovirus*, and *Ranavirus*, can inhibit the synthesis of host proteins, RNA, and DNA, as well as regulating cell apoptosis, modulating host immune responses, and exploiting various intracellular signaling pathways to facilitate viral replication and survival^1^. However, the infection mechanism employed by megalocytiviruses is largely unknown. Nevertheless, some megalocytivirus genes have been found to encode functional proteins that inhibit the activation of nuclear factor-κB and interferon (IFN)-γ, impair host integrin-linked kinase signaling, suppress cytokine signaling, and induce cell apoptosis^4^,^5^,^6^,^7^,^8^,^9^.

MicroRNAs (miRNAs) play important roles in the post-transcriptional regulation of gene expression. They have been linked to cellular differentiation, innate immunity, apoptosis, and oncogenic transformation, as well as many other cell-fate ‘decisions’^10^. Evidences indicate that miRNAs may be potentially ideal mediators, allowing for viruses to escape the host immune system^10^,^11^. Viral miRNAs can contribute to immune evasion indirectly, by lowering the levels of viral proteins and consequently reducing antigenicity, or directly, by suppressing components of the host immune response, such as factors that promote apoptosis or help to recruit immune effector cells to virus-infected cells^12^,^13^,^14^,^15^,^16^,^17^. Importantly, viruses have also evolved the ability to degrade, boost, or hijack cellular miRNAs to facilitate viral infection^18^,^19^,^20^. However, most knowledge of miRNAs has been gained through studies of mammalian models, and studies of both host and viral miRNAs in teleosts, especially at the functional level, are very limited.

Japanese flounder (*Paralichthys olivaceus*) is a teleost species with high economic values that is widely farmed in China and other countries. In our previous study, we discovered 121 differently expressed flounder miRNAs associated with megalocytivirus infection using high-throughput sequencing^21^. Bioinformatics analysis showed that the target genes of these miRNAs were grouped mainly into the categories of immune response, signal transduction, and apoptotic process. In the present study, we identified one of these miRNAs, pol-miR-731, that was closely associated with the early stage of megalocytivirus infection. We further investigated the role of pol-miR-731 in viral replication, the Type I interferon response, cell apoptosis, and cell cycle arrest.

## Results

### Expression profiles of flounder miRNAs during the early stage of megalocytivirus infection

**Figure 1:**
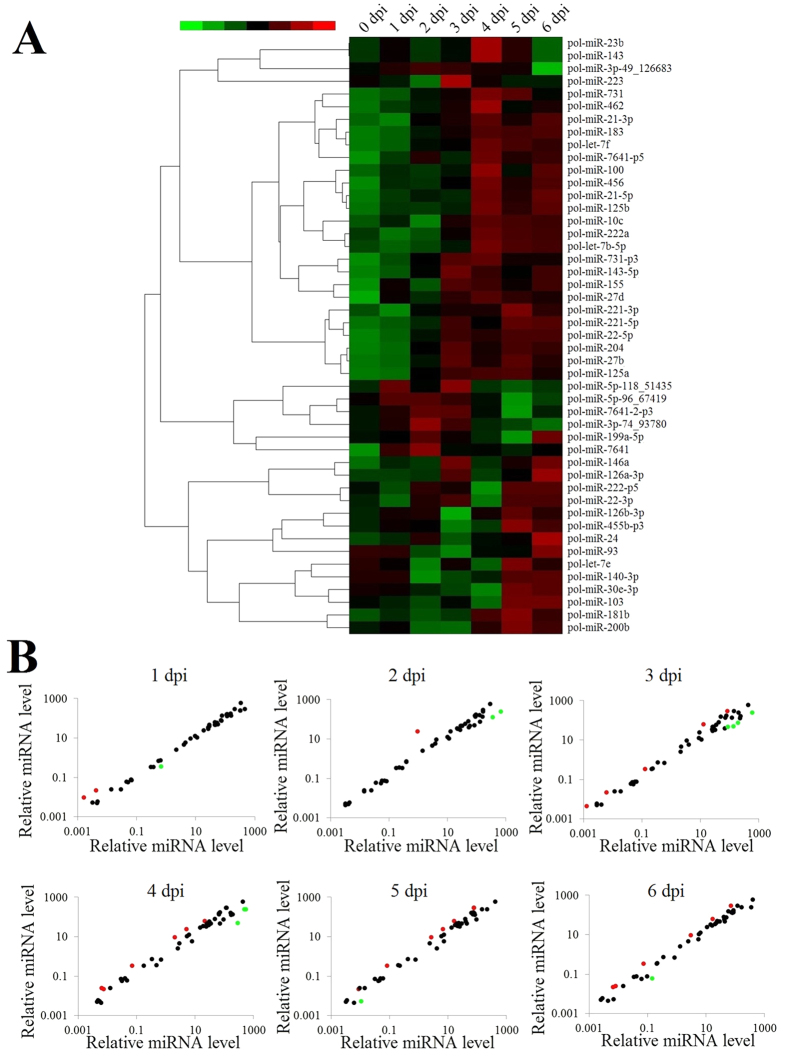
Expression profiles of flounder miRNAs during the early stage of megalocytivirus RBIV-C1 infection. (**A**) Expression levels of 47 flounder miRNAs at 0–6 days post-viral infection (dpi) are shown in different colors. Each horizontal color bar represents one miRNA, with the name indicated on the right of the bar. (**B**) Scatter plot of expression levels of 47 flounder miRNAs at 1-6 dpi compared with 0 dpi. Red and green spots represent miRNAs that were significantly (*P* < 0.01 and >2 fold) upregulated and downregulated, respectively (the names of these miRNAs are indicated in Table S1).

In a previous study, we identified 219 differentially expressed host miRNAs (≥1.2 fold and *P* < 0.05) in flounder infected with megalocytivirus for 14 days (2 dpi, 6 dpi, 10 dpi, and 14 dpi). In this and previous studies, 1 to 7 dpi was defined as the early stage of megalocytivirus infection, in which the fish showed no apparent clinical signs. To investigate the roles of flounder miRNAs in the early stage of megalocytivirus infection, we selected 47 miRNAs upregulated (≥1.2 fold and >500 copies) at 2 dpi and 6 dpi and examined their expression profiles during megalocytivirus infection at more close-spaced time points (0 dpi, 1 dpi, 2 dpi, 3 dpi, 4 dpi, 5 dpi, and 6 dpi). The results showed that the miRNAs exhibited dynamic changes in expression (Fig. 1A). Five main types of expression patterns were identified, including expression increased/decreased with time, expression increased/decreased first and then decreased/increased, and diphasic expression. The expression levels of 17 miRNAs were significantly (≥2 folds and *P* < 0.01) altered at 1 dpi to 6 dpi compared to 0 dpi. Of these miRNAs, 8 were upregulated and 9 were downregulated (Fig. 1B; Table S1).

### Effect of flounder miRNA on viral replication

**Figure 2:**
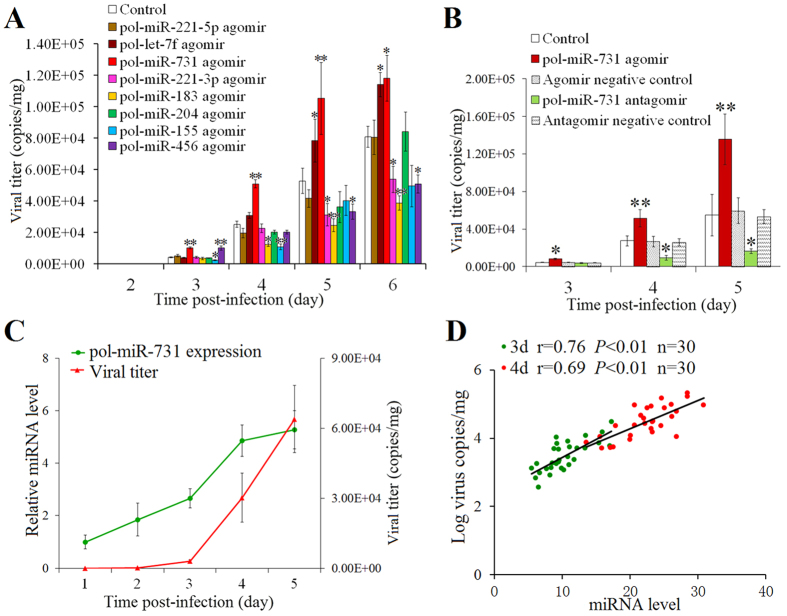
Effects of flounder miRNAs on megalocytivirus RBIV-C1 replication. (**A**) Flounders were infected with megalocytivirus in the presence or absence (control) of the agomirs of 8 flounder miRNAs, and viral copies in the spleen were determined at various time points. (**B**) Flounders were infected with megalocytivirus in the presence or absence (control) of pol-miR-731 agomir, pol-miR-731 antagomir, agomir negative control, or antagomir negative control, and viral copies in the spleen were determined at 3, 4band 5 days post-infection (dpi). (**C**) Flounders were infected with megalocytivirus and pol-miR-731expression and viral numbers in spleen were determined at different dpi. (**D**) Positive correlations between viral replication and pol-miR-731 expression level were determined at 3 d post-viral infection (dpi) (green spots) and 4 dpi (red spots). All experiments were performed three times, and values are shown as means ± SEM. *P < 0.05, **P < 0.01.

Of the 8 miRNAs significantly upregulated at the early infection stage, we examined their effects on viral replication. For this purpose, flounders were infected with megalocytivirus in the presence or absence of the agomir of each of the 8 miRNAs, and viral replication in spleen at 1 dpi to 6 dpi was determined. The results showed that pol-miR-183, pol-miR-155, and pol-miR-221-3p significantly inhibited viral replication from 4 dpi to 6 dpi, 3 dpi to 6 dpi (except for 5 dpi), and 5 dpi to 6 dpi respectively; pol-miR-731 and pol-let-7f significantly enhanced viral replication from 3 dpi to 6 dpi and 5 dpi to 6 dpi, respectively; pol-miR-456 significantly promoted viral replication at 3 dpi and reduced viral replication at 5 dpi and 6 dpi; pol-miR-204 and pol-miR-221-5p had no effect on viral replication (Fig. 2A). pol-miR-731 caused 2.0–2.5-fold increase in virus titer from 3 dpi to 5 dpi, and pol-miR-456 caused 2.4 fold increase in virus titer at 3 dpi. Conversely, pol-miR-155 suppressed viral replication by 3.1-fold at 4 dpi, and pol-miR-183 suppressed viral replication by 2.1–2.6 fold from 4 dpi to 6 dpi. Since pol-miR-731 strongly increased viral replication, we further compared the effects of its agomir and antagomir on viral replication. The results indicated that in contrast to pol-miR-731 agomir, pol-miR-731 antagomir significantly reduced viral replication by 2.8 fold and 3.3 folds at 4 dpi, and 5 dpi, respectively (Fig. 2B). No apparent effect was observed with agomir negative control or antagomir negative control.

### pol-miR-731 expression correlates with viral replication

qRT-PCR analysis showed that the level of pol-miR-731 increased with time during megalocytivirus infection, and rose markedly from 3 to 4 dpi (Fig. 2C). Likewise, after a lag at 1–3 dpi, viral replication increased dramatically from 3 to 5 dpi. There were significant positive correlations between pol-miR-731 levels and viral numbers at 3 and 4 dpi (Fig. 2D).

### pol-miR-731 specifically suppresses the expression of IRF7 and p53

**Figure 3:**
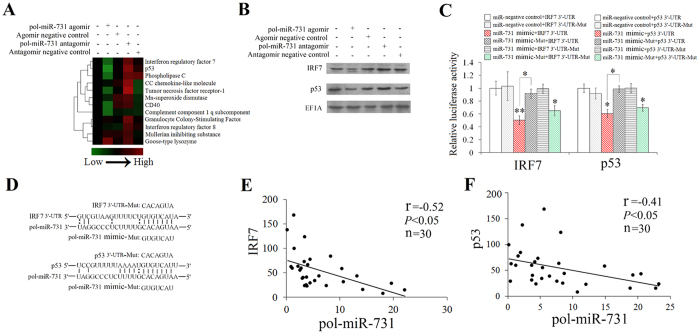
Effect of pol-miR-731 on expression of putative target genes. (A) Flounders were infected with megalocytivirus RBIV-C1 in the presence of pol-miR-731agomir, pol-miR-731 antagomir, agomir negative control, or antagomir negative control, and the expression levels of 11 potential target genes in spleen were determined by q RT-PCR. (**B**) Flounders were infected with megalocytivirus as in (**A**), and proteins prepared from the spleen were analyzed by immunoblot with antibodies against recombinant IRF7, p53, or EF1A. (**C**) 293T cells were co-transfected with pol-miR-731 mimic, pol-miR-731mimic-Mut, and different reporter plasmids (pMIR-IRF7 3′-UTR, pMIR-p53 3′-UTR, pMIR-IRF7 3′-UTR-Mut, and pMIR-p53 3′-UTR-Mut). Relative luciferase activity was measured after 24 h of transfection. The experiment was performed three times, and values are shown as means ± SEM.*P < 0.05, **P < 0.01. (**D**) Schematic diagrams showing pol-miR-731 target sites on the 3′-UTR regions of IRF7 and p53. (**E,F**) Flounders were infected with megalocytivirus, and correlations between mRNA levels of pol-miR-731 and IRF7 (**E**) and p53 (**F**) were examined by Spearman analysis. Values of correlation coefficient (r) and *P* are shown.

In our previous study, *in silico* analysis showed that pol-miR-731 potentially targeted 11 flounder genes, i.e. CC chemokine-like molecule, CD40, complement component 1 q subcomponent gamma polypeptide, goose-type lysozyme, granulocyte colony-stimulating factor, IRF7, IRF8, Mn-superoxide dismutase, Mullerian inhibiting substance, p53, phospholipase C, and tumor necrosis factor receptor-1^21^. To identify the actual target of pol-miR-731, flounders were infected with megalocytivirus in the presence of pol-miR-731 agomir or pol-miR-731 antagomir, and the expression levels of all potential target genes in spleen were determined by qRT-PCR. pol-miR-731 agomir significantly inhibited the expression of IRF7 and p53 but had no apparent effect on the expression of other genes (Fig. 3A). In contrast, pol-miR-731 antagomir significantly upregulated the expression of IRF7 and p53 (Fig. 3A). Consistently, western blot showed that pol-miR-731 agomir and antagomir reduced and increased, respectively, protein levels of IRF7 and p53 (Fig. 3B).

To examine whether the above observed effect of pol-miR-731 resulted from direct interaction between the miRNA and the 3′UTRs of IRF7 and p53, the reporter plasmids pMIR-IRF7 3′-UTR and pMIR-p53 3′-UTR were created, containing firefly luciferase reporter linked to the 3′-UTRs of IRF7 and p53 respectively. pol-miR-731 mimic and the reporter plasmid were co-transfected into 293T cells. Luciferase activity was significantly reduced in cells transfected with pol-miR-731 mimic plus pMIR-IRF7 3′-UTR or pMIR-p53 3′-UTR (Fig. 3C). In contrast, luciferase activities in 293T cells co-transfected with the reporter plasmids and pol-miR-731 mimic-Mut, a mutated pol-miR-731 mimic, were comparable to control cells (Fig. 3C). We further investigated the specificity of pol-miR-731 for IRF7 and p53 using the plasmids pMIR-IRF7 3′-UTR-Mut and pMIR-p53 3′-UTR-Mut, containing mutated IRF7 3′-UTR and p53 3′-UTR, in which the sequences of IRF7 3′-UTR and p53 3′-UTR complementary to the seed region of pol-miR-731 were altered (Fig. 3D). No significant change in luciferase activity was detected in 293T cells transfected with pol-miR-731 mimic plus pMIR-IRF7 3′-UTR-Mut or pMIR-p53 3′-UTR-Mut, compared with the control cells (Fig. 3C).

In addition to the above *in vitro* studies, we also compared the *in vivo* expression levels of pol-miR-731 and its target genes during viral infection. Expression levels of pol-miR-731 were negatively correlated with expression levels of IRF7 (r = −0.52 and *P* < 0.05) and p53 (r = −0.41 and *P* < 0.05) in flounders infected with megalocytivirus (Fig. 3E,F).

### pol-miR-731 disrupts megalocytivirus-induced Type I IFN response

**Figure 4:**
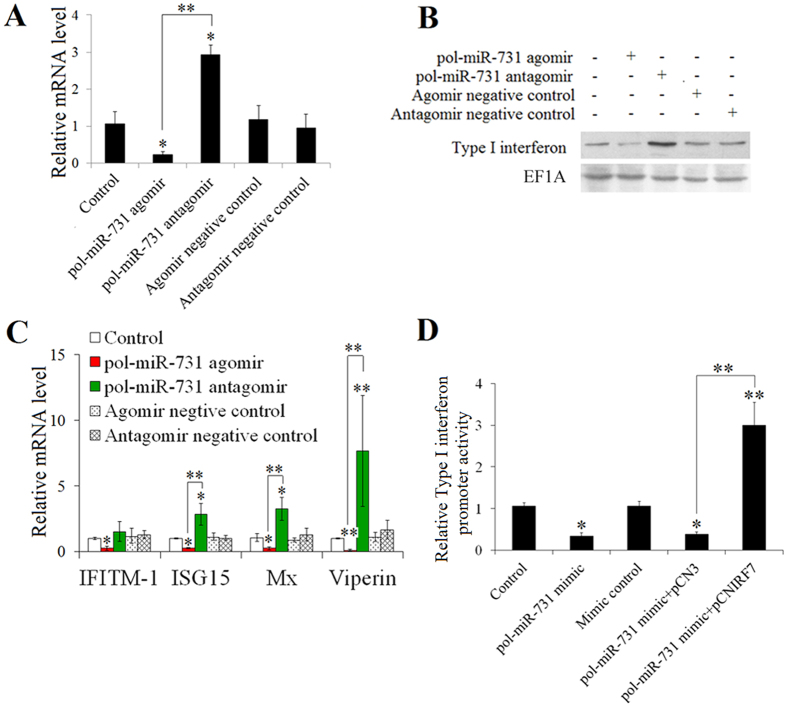
Effect of pol-miR-731 on Type I interferon (IFN) response. (**A,B**) Flounders were infected with megalocytivirus RBIV-C1 in the presence or absence (control) of pol-miR-731 agomir, pol-miR-731 antagomir, agomir negative control, or antagomir negative control, and the expression level of IFN in spleen was determined at mRNA and protein levels at 4 days post-infection (dpi) by qRT-PCR (**A**) and western blot (**B**), respectively. (**C**) mRNA levels of IFN-stimulated genes in the above samples were determined by qRT-PCR. (**D**) FG-9307 cells were transfected with or without (control) pGL3-IFNp plus pol-miR-731 mimic, mimic control, pol-miR-731 mimic + pCNIRF7, or pol-miR-731 + pCN3; the cells were treated with poly I:C, and luciferase activity was measured at 24 h post treatment. All experiments were perforemed three times, and values are shown as means ± SEM. *P < 0.05, **P < 0.01.

IRF7 is an interferon regulatory factor, and we therefore determined if pol-miR-731 affected the type I IFN response through IRF7. pol-miR-731 agomir and antagomir respectively inhibited and promoted IFN expression at both the mRNA and protein levels in megalocytivirus-infected flounders (Fig. 4A,B). qRT-PCR analysis of the expression of IFITIM1, ISG15, Mx, and viperin, which are known IFN-stimulated genes (ISGs) in teleosts^22^,^23^,^24^, showed that all four genes were significantly downregulated by pol-miR-731 agomir (Fig. 4C). In contrast, the expression levels of all examined ISGs, except for IFITIM1, were significantly upregulated by pol-miR-731 antagomir (Fig. 4C). To examine whether the effect of pol-miR-731 could be rescued by IRF7 overexpression, flounders were administered pol-miR-731 agomir plus the plasmid pCNIRF7, which expresses IRF7 in fish (Fig. S1), or the control plasmid pCN3. Type I interferon expression levels in fish administered pol-miR-731 agomir plus pCNIRF7 were comparable to levels in control fish and significantly higher than those in fish administered pol-miR-731 agomir alone or pol-miR-731 agomir plus pCN3 (Fig. S2A). Western blotting indicated that IFN production in fish administered pol-miR-731 agomir plus pCNIRF7 was higher than that in fish administered pol-miR-731 agomir or pol-miR-731 agomir plus pCN3 (Fig. S2A). Consistently, ISG expression levels were significantly higher in fish administered pol-miR-731 agomir plus pCNIRF7 (Fig. S2B).

To verify the specificity of pol-miR-731 for the IFN response, we co-transfected pol-miR-731 mimic into FG-9307 cells with pGL3-IFNp, a luciferase reporter of IFN promoter activity. Subsequent luciferase assay showed that pol-miR-731 mimic significantly inhibited the luciferase activity of pGL3-IFNp (Fig. 4D). However, luciferase activity was significantly increased when FG-9307 cells were transfected with pGL3-IFNp and pol-miR-731 mimic with pCNIRF7 rather than the control plasmid pCN3 (Fig. 4D).

### pol-miR-731 inhibits megalocytivirus-induced apoptosis

**Figure 5:**
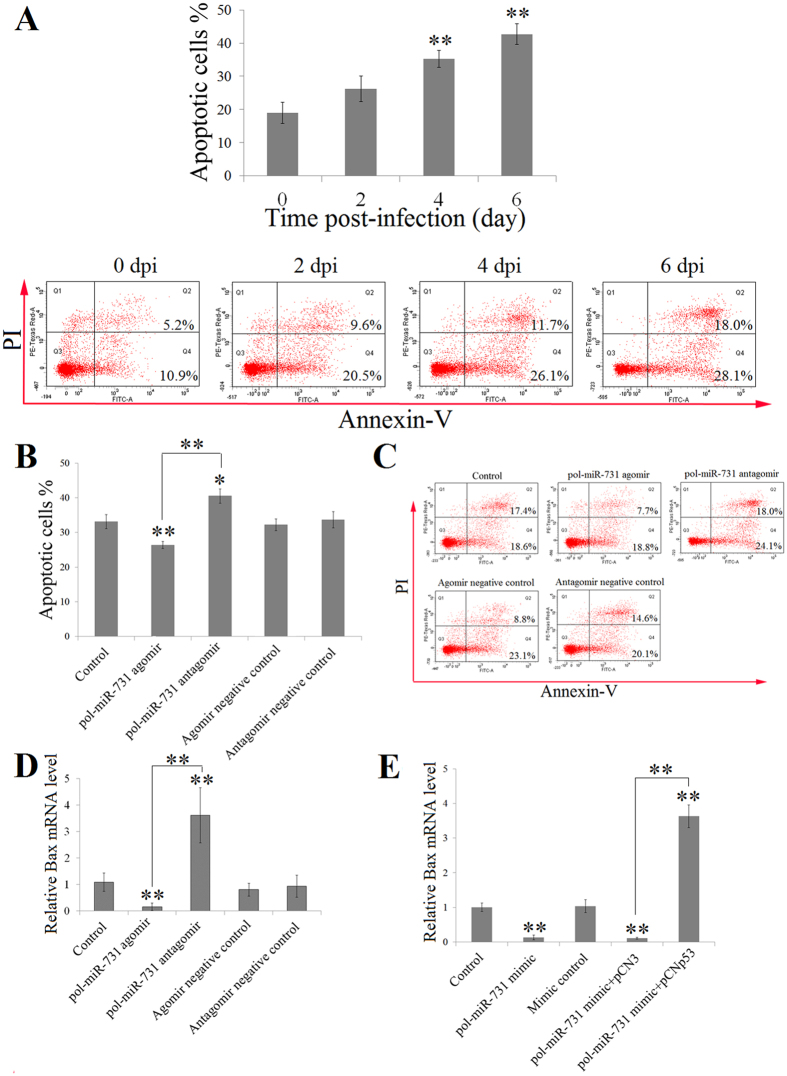
Effect of pol-miR-731 on cell apoptosis. (**A**) Flounders were infected with megalocytivirus RBIV-C1, and splenocytes apoptosis was determined by Annexin V-PI assay at various days post-infection (dpi) (upper panel). Lower panel shows the results of one representative experiment. (**B,C**) Flounders were infected with megalocytivirus in the presence or absence (control) of pol-miR-731agomir, pol-miR-731antagomir, agomir negative control, or antagomir negative control, and splenocyte apoptosis was assayed at 4 dpi using Annexin V-PI (**B**). The results of one representative experiment are shown in (**C**). (**D**) Flounders were infected with megalocytivirus as in (**A**), and expression levels of Bax were determined by qRT-PCR. (**E**) FG-9307 cells were transfected with or without (control) pol-miR-731 mimic, mimic negative control, pol-miR-731 mimic plus pCNp53, or pol-miR-731 mimic plus pCN3, treated with 1 μM doxycycline, and Bax expression was determined as above. All experiments were performed three times, and values are shown as means ± SEM.*P < 0.05, **P < 0.01.

Annexin V-PI assay revealed that apoptosis of splenocytes was significantly increased at 4 and 6 dpi in flounders infected with megalocytivirus (Fig. 5A). Given that pol-miR-731 inhibited the expression of p53, which is involved in apoptosis, we examined whether pol-miR-731 had any effect on megalocytivirus-induced apoptosis. The presence of pol-miR-731 agomir in flounders infected with megalocytivirus reduced the ratio of apoptotic splenocytes by 1.3-fold compared with control fish, whereas the presence of pol-miR-731 antagomir increased the ratio of apoptotic splenocytes by 1.2-fold (Fig. 5B,C). However, when the plasmid pCNp53, which expresses p53 in fish (Fig. S1), was co-present with pol-miR-731 agomir, splenocyte apoptosis was significantly increased to a level comparable to that in control fish (Fig. S3). The control plasmid pCN3 had no apparent effect. These results suggested that the effect of pol-miR-731 was mediated through p53. Given that Bax is a target gene of p53 and is known to be an important effector of apoptosis^25^,^26^, we determined Bax expression under different conditions. pol-miR-731 agomir significantly inhibited the expression of Bax, whereas pol-miR-731 antagomir significantly upregulated it (Fig. 5D). We further investigate the effect of pol-miR-731 *in vitro*, by transfecting FG-9307 cells with pol-miR-731 mimic or pol-miR-731 mimic plus pCNp53, and measuring Bax expression in the transfectants. Bax expression was significantly inhibited in cells transfected with pol-miR-731 mimic, but significantly increased in cells transfected with pol-miR-731 mimic plus pCNp53 (Fig. 5E).

### pol-miR-731 blocks megalocytivirus-induced cell cycle arrest

**Figure 6:**
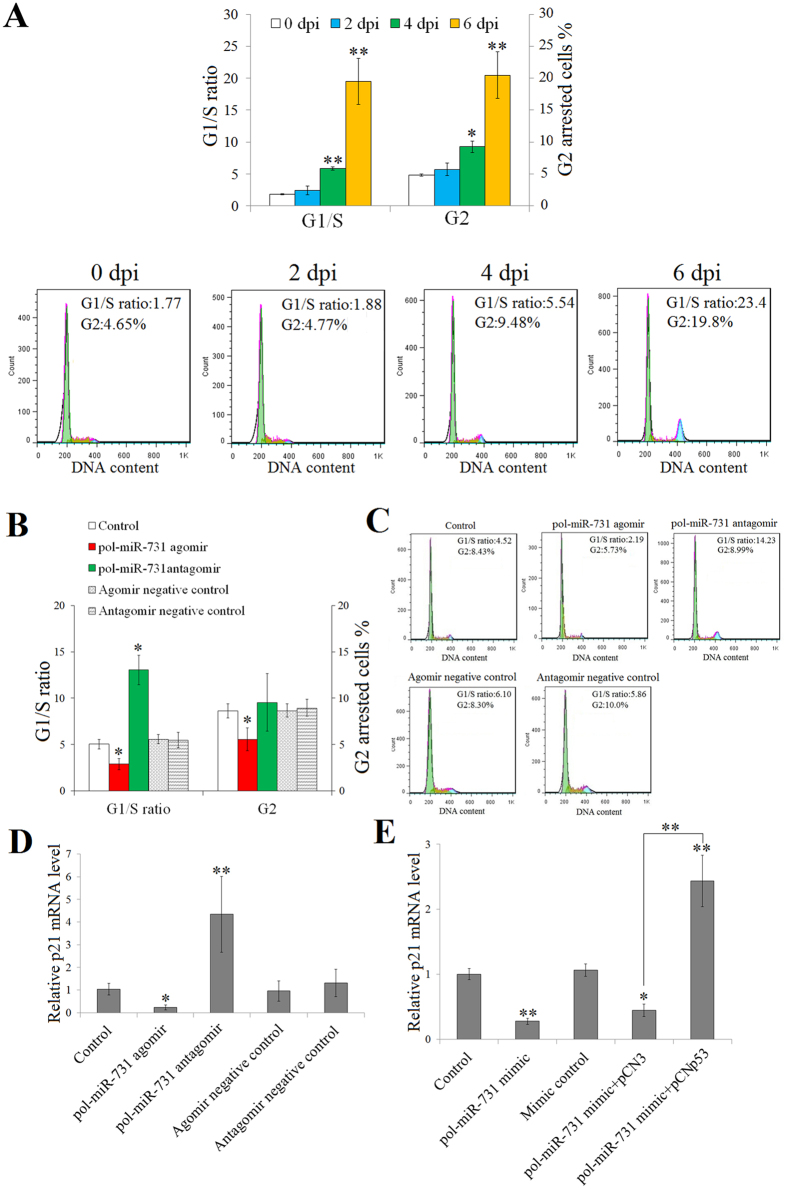
Effect of pol-miR-731 on cell cycle arrest. (**A**) Flounders were infected with megalocytivirus RBIV-C1, and splenocytes were examined for cell cycle arrest at various days post-infection (dpi) (upper panel). Lower panel shows the results of one representative experiment. (**B,C**) Flounders were infected with megalocytivirus in the presence or absence (control) of pol-miR-731agomir, pol-miR-731antagomir, agomir negative control, or antagomir negative control, and cell cycle arrest in splenocytes was assayed at 4 dpi by flow cytometry (**B**). The results of one representative experiment are shown in (**C**). (**D**) Flounders were infected with megalocytivirus as in (**B**), and expression levels of p21 were determined by qRT-PCR. (**E**) FG-9307 cells were transfected with or without (control) pol-miR-731 mimic, mimic negative control, pol-miR-731 mimic plus pCNp53, or pol-miR-731 mimic plus pCN3, treated with 1 μM doxycycline, and p21 expression levels in the transfectants were determined as above. All experiments were performed three times, and significant differences in values are indicated by asterisks. *P < 0.05, **P < 0.01.

In addition to its role in apoptosis, p53 is also a key player in cell cycle control. We therefore investigated the potential role of pol-miR-731 in the cell cycle by examining the impact of megalocytivirus infection on cell cycle progression, and showed that cell cycle arrest increased significantly as infection progressed (Fig. 6A). To examine the effect of pol-miR-731 on the cell cycle, cell cycle progression was determined in flounders infected with megalocytivirus in the presence or absence of pol-miR-731 agomir or pol-miR-731 antagomir. pol-miR-731 agomir significantly reduced the number of splenocytes that stayed in G1 and G2 phases (Fig. 6B,C), while pol-miR-731 antagomir significantly increased the number of splenocytes in G1 phase (Fig. 6B,C). To verify that the effect of pol-miR-731 was realized through p53, pCNp53 was co-administered with pol-miR-731 agomir to megalocytivirus-infected flounders. The presence of pCNp53, but not the control plasmid pCN3, counteracted the effect of pol-miR-731 agomir on cell cycle arrest (Fig. S4). qRT-PCR analysis indicated that pol-miR-731 agomir significantly inhibited p21 expression, which is known to be regulated by p53 and is essential to cell cycle arrest^25^,^27^, whereas pol-miR-731 antagomir significantly upregulated p21 expression (Fig. 6D). Consistent with the above *in vivo* results, *in vitro* analysis indicated that p21 expression was significantly downregulated in FG-9307 cells transfected with pol-miR-731 mimic, but significantly upregulated in cells transfected with pol-miR-731 mimic plus pCNp53 (Fig. 6E).

### Overexpression of IRF7 and p53 abrogates the effect of pol-miR-731 on viral replication

**Figure 7:**
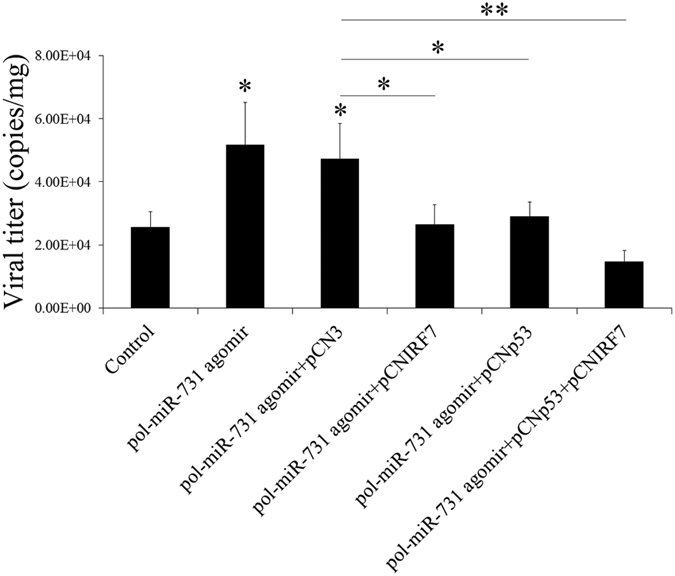
Effect of IRF7 and p53 overexpression on pol-miR-731-promoted viral replication. Megalocytivirus RBIV-C1-infected flounders were administered with or without (control) pol-miR-731agomir, or with pol-miR-731agomir plus pCN3, pCNIRF7, pCNp53, or pCNIRF7 + pCNp53. The amounts of virus in the spleen were determined at 4 days post-infection. The experiment was performed three times, and significant differences in values are indicated by asterisks. *P < 0.05, **P < 0.01.

As noted above, pol-miR-731 promoted megalocytivirus replication. To determine if this effect of pol-miR-731 was dependent on IRF7 and p53, megalocytivirus-infected flounders were treated with pol-miR-731 agomir in the presence of pCNIRF7, pCNp53, or both pCNIRF7 and pCNp53. Compared with viral numbers in fish treated with pol-miR-731 agomir alone, numbers in fish treated with pol-miR-731 agomir plus pCNIRF7, pCNp53, and pCNIRF7 + pCNp53 were significantly reduced to levels similar to those in untreated fish (Fig. 7). In contrast, viral numbers in fish treated with pol-miR-731 agomir plus the control plasmid pCN3 were comparable to those in fish treated with pol-miR-731 agomir.

## Discussion

Host miRNA expression is profoundly influenced by viral infection, providing a potential mechanism for host antiviral defense, or for viral manipulation of the host system^28^,^29^. Recent studies in higher vertebrates showed that viruses have evolved various strategies to evade or utilize host miRNAs to benefit the viral life cycle^10^,^18^. For example, herpesvirus saimiri and mouse cytomegalovirus degrade host miRNA miR-27 to facilitate viral replication^30^, and hepatitis C virus can hijack miR-122 to regulate viral replication^31^. Meanwhile, in invertebrate, some studies also found that virus (such as ascovirus) can manipulate host miRNAs expression to facilitate viral colonization^32^. Thus, it is believed that miRNA-mediated regulatory mechanism is an effective way for virus to persist in host^33^,^34^,^35^. However, no similar studies have been carried out in lower vertebrates, including teleosts. We previously identified 59 host miRNAs that were differentially expressed in flounders infected with megalocytivirus for 1–6 days, whereas viral miRNAs were scarce^21^, raising the interesting question of whether host miRNAs may be involved in the early stage of viral infection, as observed with human cytomegalovirus, which employs host miRNAs to promote viral latency^33^. In this study, we therefore investigated eight flounder miRNAs that were significantly upregulated in the early stage of megalocytivirus infection, among which pol-miR-731 was found to promote viral replication most effectively at 3–5 dpi *in vivo*. To the best of our knowledge, this is the first observation of a host miRNA induced by a virus serving as a positive regulator of viral replication in teleosts. qRT-PCR analysis indicated that pol-miR-731 expression in flounders was significantly stimulated by megalocytivirus at 4 and 5 dpi, similar to a previous observation of miR-731 expression in rainbow trout infected with viral hemorrhagic septicemia virus^36^. These observations suggest that miR-731 may play an important role in the early stage of viral infection.

Type I IFN response is the principal response that mediates antiviral innate immunity^37^,^38^,^39^. To date, 36 Type I IFN-regulated miRNAs have been reported in mammalian systems, targeting genes encoding components of Type I IFN pathway^40^. Porcine reproductive and respiratory syndrome virus is known to promote infection by upregulating miR-30c, which impairs IFN signaling^41^, and enterovirus-71 evades the host innate immune response by downregulating the expression of miR-526a, which positively regulates Type I IFN production^42^. Kaposi’s sarcoma-associated herpesvirus encodes miR-K12-11, which attenuates the IFN response by decreasing I-kappa-B kinase (IKKε)-mediated IRF3/IRF7 phosphorylation and inhibiting the activation of IKKε-dependent ISGs^43^. Although IRF7 is a master regulator of the type-I IFN response, there is no documented evidence of miRNAs directly targeting IRF7. In the current study, we found that pol-miR-731 agomir and pol-miR-731 antagomir significantly downregulated and upregulated, respectively, the expression of IRF7 *in vivo* at both the mRNA and protein levels, and demonstrated a negative correlation between pol-miR-731 and IRF7 expression levels. Under *in vitro* conditions, pol-miR-731 mimic inhibited transcription of the luciferase reporter gene linked to the 3′-UTR of IRF7, but only if the 3′-UTR sequence complementary to the seed region of pol-miR-731 was intact. These results suggest that pol-miR-731 negatively regulates IRF7 by direct and specific interaction with the 3′-UTR of IRF7. In line with this conclusion, qRT-PCR analysis indicated that the expression of IFN and ISGs, which were induced by megalocytivirus, were significantly downregulated and upregulated by pol-miR-731 agomir and pol-miR-731 antagomir, respectively, while overexpression of IRF7 abolished the effect of pol-miR-731 on IFN and ISGs. Furthermore, pol-miR-731 mimic reduced the promoter activity of IFN in transfected flounder cells *in vitro*, whereas overexpression of IRF7 rescued the inhibition caused by pol-miR-731 mimic. Overall, these results indicate that pol-miR-731 inhibits the megalocytivirus-triggered type I IFN response by targeting IRF7, which represents a novel regulatory mechanism of virus-induced miRNA in teleost. It is possible that miR-731, like mammalian miR-146a and miR-155 that block type I IFN signaling^44^,^45^, may negatively regulate the Type I IFN response as a part of the host protection mechanism to restrict the Type I IFN response at an appropriate level and duration.

Teleost miR-731 molecules are orthologs of mammalian miR-425 and share the same seed sequence^36^. miR-425 is known to be involved in gastric carcinogenesis, and inhibition of miR-425 in HGC-27 cells not only reduced cell proliferation and cell cycle progression but also impaired cell migration and invasion^46^. However, the working mechanism of miR-425 is obscure. We found that pol-miR-731 targeted both IRF7 and p53. As for IRF7, pol-miR-731 inhibited p53 expression in a manner that required the sequence in the 3′-UTR of p53 complementary to the seed region of pol-miR-731, suggesting that the inhibitory effect of pol-miR-731 was mediated by direct binding of the miRNA to the target gene. p53 protein plays vital roles in both apoptosis and cell cycle arrest. Previous reports have shown that miRNAs can regulate the p53 pathway to mediate virus–host interactions. In mammals, hepatitis B virus downregulates miR-122 to promote the expression of cyclin G1, which specifically interacts with p53 to abrogate p53-mediated inhibition of viral replication^47^; Kaposi sarcoma-associated herpes virus utilizes miR-K1 to target p21 and prevent cell-cycle arrest activated by the DNA-damage response through p53^48^. In teleosts, the TRAF protein encoded by ORF111L of ISKNV was shown to cause caspase 8-mediated apoptosis in mandarin fish and zebrafish^5^, though the involvement of p53 was unclear. Virus-induced cell cycle arrest has not previously been documented in teleosts. In our study, megalocytivirus infection in flounders induced apparent splenocyte apoptosis and cell cycle arrest, both of which were significantly reduced by pol-miR-731 agomir and increased by pol-miR-731 antagomir, while the inhibitory effects of pol-miR-731 agomir on apoptosis and cell cycle were counteracted by overexpression of p53. These results indicate that pol-miR-731 negatively regulates apoptosis and cell cycle arrest by suppressing p53 expression. In line with these observations, *in vitro* studies in FG-9307 cells showed that pol-miR-731 mimic significantly downregulated the expression of the p53 target genes Bax and p21, confirming the negative regulatory role of pol-miR-731 on the p53 pathway.

Viral loads in megalocytivirus-infected flounders *in vivo* were significantly increased by treatment with pol-miR-731 agomir, while viral loads were similar to those in untreated control fish when infected flounders were treated with pol-miR-731 agomir plus pCNIRF7 or pCNp53, indicating that overexpression of IRF7 or p53 completely abrogated the effect of pol-miR-731 on viral replication. These results indicate a causal link between the inhibitory effect of pol-miR-731 on IRF7/p53 and the promoting effect of pol-miR-731 on viral infection.

**Figure 8:**
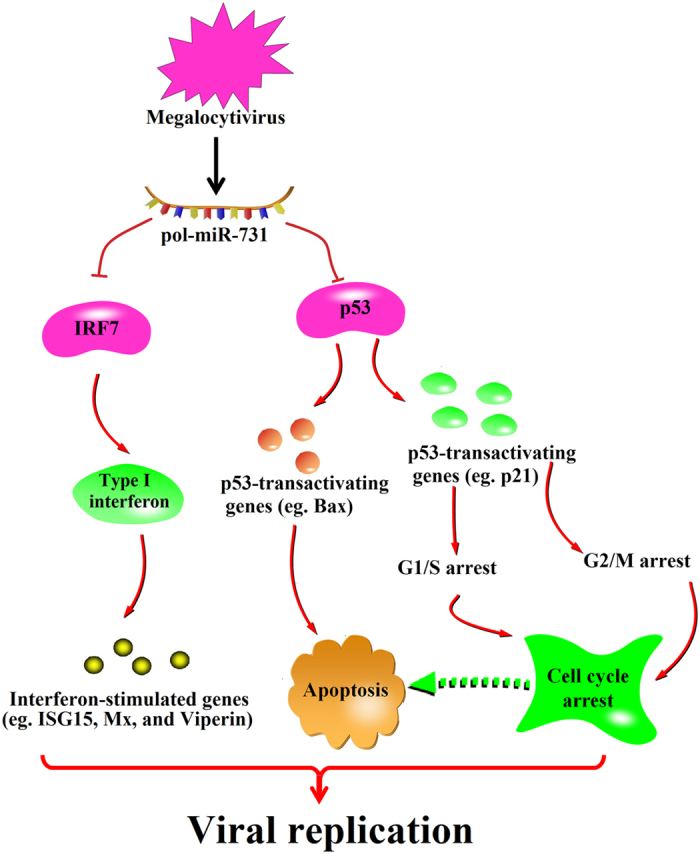
Model of the hijacked function of pol-miR-731 in megalocytivirus RBIV-C1 infection. During the early infection stage, megalocytivirus upregulates the expression of pol-miR-731, which in turn inhibits IRF7 and p53 expression, leading to restriction of Type I interferon response and inhibition of apoptosis and cell cycle arrest, respectively, thus promoting megalocytivirus infection. Arrows indicate upregulation; blunt arrows denote inhibition.

In summary, we identified a novel immune-evasion mechanism employed by megalocytivirus, which involves regulation of the expression of a host miRNA. Megalocytivirus infection upregulates miR-731 expression, which in turn suppresses the expression of IRF7 and p53. Suppression of IRF7 and p53 subsequently leads to inhibition of the type I interferon response, cellular apoptosis, and cell cycle arrest, resulting in increased viral replication during the early stage of infection. Based on these observations, we propose a model delineating the role of pol-miR-731 in virus–host interaction (Fig. 8). These results provide the first evidence for a teleost miRNA exerting profound impacts on multiple biological processes by negatively regulating both IRF7- and p53-mediated pathways. Furthermore, the natural inhibitory function of a host miRNA is shown to be exploited by a virus to counteract the host antiviral immune defense mechanism and facilitate viral infection.

## Materials and Methods

### Ethics statement

All protocols for experiments involving live animals conducted in this study were approved by the Ethics Committee of the Institute of Oceanology, Chinese Academy of Sciences (Shandong, China), and the experiments were carried out in accordance with the approved protocols.

### Fish maintenance

Clinically healthy Japanese flounder (weighing about ~13.6 g) were purchased from a local fish farm and maintained at 22 °C in aerated seawater. Fish were acclimatized in the laboratory for 2 weeks before the experiments and were confirmed to be pathogen-free by examination of bacteria and megalocytivirus in the blood, liver, kidney, and spleen of randomly sampled fish, as reported previously^49^. For tissue collection, fish were euthanized with an overdose of tricaine methanesulfonate (Sigma, St. Louis, MO, USA).

### Quantitative real-time reverse transcription PCR (qRT-PCR)

To examine miRNA expression, miRNAs were extracted from spleen and reverse transcripted, and qRT-PCR was carried out as reported previously^21^. The expression of pol-miR-371 target genes and immune genes in spleen were investigated by qRT-PCR, elongation factor-1-α (EF1A) as an internal control^50^. The relative mRNA levels of target genes were transformed into log values, which were used to draw the heat map with Java TreeView v1.1.6 software (Alok Saldanha, Sourceforge.net, USA). All experiments were performed three times.

### Antibody preparation and western blot

To prepare recombinant IRF7 (rIRF7) and p53 (rp53), the coding sequences of flounder IRF7 and p53 were amplified by PCR with primers F1/R1 and F2/R2, respectively (Table S1), and the PCR products were inserted into pET259^51^ at the SwaI site. The proteins were expressed, purified, and cleared of endotoxin as reported previously^51^. Mouse antibodies against rIRF7 and rp53 were prepared as reported previously^51^ and purified using ProteinA/G Beads (Solarbio, Beijing, China).

IRF7 and p53 production in flounder spleen were examined by western blotting. Flounders were infected with megalocytivirus as described above for 4 days, after which the spleen protein were prepared as reported previously^51^. Then, proteins were subjected to 12% SDS-PAGE and transferred onto polyvinylidene difluoride membranes (Millipore, Watford, UK). The following steps were carried out according to previous study^51^, of which the mouse antibodies against rPoIRF7 (1/4000 dilution) or rPop53 (1/2000 dilution) were used. Finally, protein bands were visualized using enhanced chemiluminescence Western Blotting Substrate (Promega, Madison, WI, USA).

### miRNA mimic, agomir, and antagomir

pol-miR-731 mimic and pol-miR-731 mimic-Mut, which is pol-miR-731 mimic with the seed sequence mutated to its complementary sequence, were synthesized by RiboBio (Guangzhou, China). The negative control, mimic control, was designed and synthesized by the same company. miRNA agomirs and antagomirs are chemically engineered oligonucleotides especially suitable for *in vivo* use. pol-miR-731 agomir and pol-miR-731 antagomir and their negative controls were synthesized by Genepharma (Shanghai, China). The sequences of the mimic, mimic control, agomir, and antagomir used in these studies have been listed in supplementary Table S2.

### Plasmid construction

The plasmids pMIR-IRF7 3′-UTR and pMIR-p53 3′-UTR contain the firefly luciferase gene with its 3′-untranslated region (UTR) replaced by the 3′-UTRs of IRF7 and p53, respectively. The 3′ UTR of flounder IRF7 and the 3′-UTR of flounder p53 were amplified by PCR with the primer pairs F3/R3 and F4/R4 (Table S3), respectively. The PCR products were inserted into the luciferase vector pMIR-REPORTER (AmBio, Life Technologies, USA) at the Spe I/Hind III sites, resulting in pMIR-IRF7 3′-UTR and pMIR-p53 3′-UTR, respectively. pMIR-IRF7 3′-UTR-Mut and pMIR-p53 3′-UTR-Mut are identical to pMIR-IRF7 3′-UTR and pMIR-p53 3′-UTR, respectively, except that the sequences of the 3′-UTRs of IRF7 and p53 complementary to pol-miR-731 seed sequence were mutated to their complementary sequences by overlap PCR. To construct pGL3-IFNp, the promoter region of flounder Type I IFN was amplified (634 bp to -1 bp) with primers F5/R5 (Table S3), and the PCR product was inserted into the Mlu I/Hind III sites of pGL3 (Promega, Madison, USA), a luciferase reporter vector providing a basis for the quantitative analysis of factors that potentially regulate gene expression. To construct pCNIRF7 and pCNp53, which were designed to express flounder IRF7 and p53, respectively, in fish, the coding sequences of flounder IRF7 and p53 were amplified with primer pairs F1/R1 and F2/R2 (Table S3), respectively, and inserting the PCR products into the eukaryotic expression vector pCN3^22^ at the EcoRV site. All plasmids were confirmed by sequencing.

### Cell culture and transfection

FG-9307, a cell line established from Japanese flounder gill cells^52^, were cultured at 24 °C in L-15 (Thermo Scientific HyClone, USA) containing 20% fetal bovine serum (FBS) (Gibco, Invitrogen Corp., Carlsbad, USA). 293T human embryonic kidney epithelial cells (CBTCCCAS, Shanghai, China) were cultured in DMEM (Invitrogen, Grand Island, USA) supplemented with 10% FBS in a humidified atmosphere of 37 °C and 5% CO_2_. Flounder splenocytes were prepared by removing the spleen aseptically, washing with PBS, and dicing. Spleen cells were released by trituration using 100 μm nylon tamis cellulaire (BD Falcon, USA). Erythrocytes in the cell preparation were lysed with Red Blood Cell Lysis Buffer (Solarbio, Beijing, China) three times on ice. The splenocytes were then resuspended in L15 medium containing 10% FBS.

Transfection was performed as reported previously^53^. The cells were co-transfected with 500 ng of plasmid (pMIR-IRF7 3′-UTR, pMIR-p53 3′-UTR, pMIR-IRF7 3′-UTR-Mut, or pMIR-p53 3′-UTR-Mut) and 20 pmol of the synthesized miRNA (pol-miR-731 mimic, pol-miR-731 mimic-Mut, or pol-miR-731 mimic control) using Lipofectamine™ 2000 (Invitrogen, Carlsbad, USA) according to the manufacturer’s instructions. Transfection efficiency was monitored by co-transfection with 200 ng pMIR-REPORT β-gal control vector (Promega, Madison, USA). Different cultures of FG-9307 cells were transfected using Lipofectamine 2000 as follows: (i) cells were transfected with 30 pmol pol-miR-731 mimic or mimic control to determine the effect of pol-miR-731 on cell apoptosis and cell cycle arrest; (ii) cells were co-transfected with 30 pmol pol-miR-731 mimic and 200 ng pCN3 or pCNp53 to determine the effect of pol-miR-731 on apoptosis and cell cycle arrest through p53; (iii) cells were co-transfected with 300 ng pGL3-IFNp, 200 ng pSV-β-Galactosidase control vector, and 30 pmol pol-miR-731 mimic or mimic control to determine the effect of pol-miR-731 on IFN response; or (iv) cells were transfected with 30 pmol pol-miR-731 mimic mixed with 300 ng pGL3-IFNp, 200 ng pSV-β-Galactosidase control vector, and 200 ng pCNIRF7 or pCN3 to determine the dependence of the pol-miR-731 activity on IRF7.

### Luciferase reporter assays

To determine the interaction between pol-miR-731 and 3′ UTRs of flounder IRF7 and p53, 293T cells were transfected with plasmids and miRNA as described above for 24 h. The cells were then lysed, and luciferase activity was measured using a Dual-Light reporter gene assay system (Life Technologies, USA) according to the manufacturer’s instructions. The results of the luciferase activity were corrected by subtracting the values for β-galactosidase. To determine the IFN promoter activity, FG-9307 cells were transfected with pGL3-IFNp as above for 12 h, followed by addition of poly I:C (50 μg/ml) and incubation at 24 °C for 24 h^54^. Luciferase and β-galactosidase activities were measured as above. All assays were performed three times.

### *In vivo* infection

To examine the expression profiles of flounder miRNAs during megalocytivirus infection, flounders were injected intraperitoneally (i.p.) with megalocytivirus RBIV-C1 (10^5^ copies/fish) as described previously^21^. Fish (three at each time point) were euthanized at 0, 1, 2, 3, 4, 5, and 6 days post-infection (dpi), and spleens were collected under aseptic conditions and used for examination of miRNA expression and determination of viral loads, as reported previously^21^. To determine the effect of the agomirs of 8 flounder miRNAs on viral replication, flounders were injected i.p. with megalocytivirus (10^5^ copies/fish) plus each of the miRNA agomirs (2μg agomir/1 g fish)^53^. The fish were re-injected with miRNA agomirs at 1 and 3 dpi to maintain the *in vivo* level of the agomir, as reported previously^55^. Spleens were removed from the fish (5/time point) at 1–6 dpi, and used to determine viral copies as above. To examine the effect of pol-miR-731, flounders were infected with megalocytivirus plus pol-miR-731-agomir, pol-miR-731-antagomir, agomir negative control, or antagomir negative control as above, and viral copies in the spleen were determined at 4 dpi, as above. To examine the dependence of pol-miR-731 activity on IRF7 and p53, flounders were injected as above with megalocytivirus plus pol-miR-731-agomir and pCN3, pCNIRF7, or pCN53 (1 μg plasmid/1 g fish) and viral copies in the spleen were determined at 4 dpi, as above. All experiments were performed three times.

### Flow cytometry analysis of apoptosis and cell cycle

To measure apoptosis, flounder splenocytes and FG-9307 cells were treated with fluorescein isothiocyanate (FITC)-conjugated annexin V and propidium iodide (PI) for 15 min in the dark according to the manufacturer’s instructions (Majorbio Biotech, Shanghai, China). The cells were then subjected to flow cytometry using a FACSort Flow Cytometer (BD Biosciences, USA). To assess cell cycle arrest, FxCycle PI/RNase Staining Solution (Thermo Fisher Scientific Inc., USA) was used for flow cytometric analysis of the DNA content in fixed cells, according the manufacturer’s instructions. Data analysis was performed using FlowJo software 7.6.1 (Tree Star Inc, Ashland, OR, USA).

### Statistical analysis

Statistical analyses were performed using analysis of variance (ANOVA) in GraphPad Prism 6 (GraphPad Software, Inc. La Jolla, CA, USA).

### Importance

Megalocytivirus is a severe fish pathogen. However, the infection mechanism employed by megalocytivirus, especially in relation to immune evasion, remains to be investigated. In this study, we identified a miRNA, pol-miR-731, encoded by Japanese flounder as a target regulated by megalocytivirus to facilitate viral infection. We observed that pol-miR-731 was upregulated by megalocytivirus at the early infection stage and promoted viral replication. pol-miR-731 exerted its effect by direct repression of IRF7 and p53 expression, leading to inhibition of Type I interferon response, apoptosis, and cell cycle arrest. Our study thus revealed a novel immune evasion mechanism of megalocytivirus, and provides the first evidence for the regulation of several biological processes vital to viral infection by a host miRNA. These results further our current understanding of the infection strategy of megalocytivirus and also add new insights into the function of miRNAs in lower and higher vertebrates.

## Additional Information

**How to cite this article**: Zhang, B.-c. *et al*. pol-miR-731, a teleost miRNA upregulated by megalocytivirus, negatively regulates virus-induced type I interferon response, apoptosis, and cell cycle arrest. *Sci. Rep.* **6**, 28354; doi: 10.1038/srep28354 (2016).

## Supplementary Material

Supplementary Information

## References

[b1] Chinchar V. G., Hyatt A., Miyazaki T. & Williams T. Family Iridoviridae: poor viral relations no longer. Curr Top Microbiol 328, 123–170 (2009). 10.1007/978-3-540-68618-7_4 19216437

[b2] Zhang B. C., Zhang J. & Sun L. Streptococcus iniae SF1: complete genome sequence, proteomic profile, and immunoprotective antigens. Plos One 9, ARTN e91324 10.1371/journal.pone.0091324 (2014). PMC3951389 24621602

[b3] Kurita J. & Nakajima K. Megalocytiviruses. Viruses-Basel 4, 521–538, 10.3390/v4040521 (2012). PMC3347321 22590684

[b4] Wang R. . ORF005L from infectious spleen and kidney necrosis virus is located in the inner mitochondrial membrane and induces apoptosis. Virus Genes 49, 269–277, 10.1007/s11262-014-1088-2 (2014). 24862228

[b5] He B. L. . The viral TRAF protein (ORF111L) from infectious spleen and kidney necrosis virus interacts with TRADD and induces caspase 8-mediated apoptosis. Plos One **7**, ARTN e37001 10.1371/journal.pone.0037001 (2012). PMC3352826 22615868

[b6] Yuan J. M. . Interaction of infectious spleen and kidney necrosis virus ORF119L with PINCH leads to dominant-negative inhibition of integrin-linked kinase and cardiovascular defects in zebrafish. J Virol 89, 763–775, 10.1128/Jvi.01955-14 (2015). 25355883 PMC4301147

[b7] Xie J. F. . RING finger proteins of infectious spleen and kidney necrosis virus (ISKNV) function as ubiquitin ligase enzymes. Virus Res 123, 170–177, 10.1016/j.virusres.2006.09.003 (2007). 17049660

[b8] Guo C. J. . The viral ankyrin repeat protein (ORF1 24L) from infectious spleen and kidney necrosis virus attenuates nuclear factor-kappa B activation and interacts with I kappa B kinase beta. J Gen Virol 92, 1561–1570, 10.1099/vir.0.031120-0 (2011). 21471317

[b9] Guo C. J. . A novel viral SOCS from infectious spleen and kidney necrosis virus: interacts with Jak1 and inhibits IFN-alpha induced stat1/3 activation. Plos One **7**, ARTN e41092 10.1371/journal.pone.0041092 (2012). PMC3402483 22844427

[b10] Cullen B. R. MicroRNAs as mediators of viral evasion of the immune system. Nat Immunol 14, 205–210, 10.1038/ni.2537 (2013). 23416678 PMC3642974

[b11] Asgari S. MicroRNA functions in insects. Insect biochemistry and molecular biology 43, 388–397, 10.1016/j.ibmb.2012.10.005 (2013). 23103375

[b12] Choy E. Y. W. . An Epstein-Barr virus-encoded microRNA targets PUMA to promote host cell survival. J Exp Med 205, 2551–2560, 10.1084/jem.20072581 (2008). 18838543 PMC2571930

[b13] Nachmani D., Stern-Ginossar N., Sarid R. & Mandelboim O. Diverse herpesvirus micrornas target the stress-induced immune ligand MICB to escape recognition by natural killer cells. Cell Host Microbe 5, 376–385, 10.1016/j.chom.2009.03.003 (2009). 19380116

[b14] Diebel K. W. . Gammaherpesvirus small noncoding rnas are bifunctional elements that regulate infection and contribute to virulence *in vivo*. Mbio 6, ARTN e01670-14 10.1128/mBio.01670-14 (2015). PMC4337559 25691585

[b15] Feldman E. R. . Virus-encoded micrornas facilitate gammaherpesvirus latency and pathogenesis *in vivo*. Mbio 5, ARTN e00981-14 10.1128/mBio.00981-14 (2014). PMC4045068 24865551

[b16] Hussain M. . West Nile virus encodes a microRNA-like small RNA in the 3′ untranslated region which up-regulates GATA4 mRNA and facilitates virus replication in mosquito cells. Nucleic Acids Res 40, 2210–2223, 10.1093/nar/gkr848 (2012). 22080551 PMC3300009

[b17] Ren Q., Huang Y., He Y., Wang W. & Zhang X. A white spot syndrome virus microRNA promotes the virus infection by targeting the host STAT. Scientific reports 5, 18384, 10.1038/srep18384 (2015). 26671453 PMC4680916

[b18] Guo Y. E. & Steitz J. A. Virus meets host microRNA: the destroyer, the booster, the hijacker. Mol Cell Biol 34, 3780–3787, 10.1128/Mcb.00871-14 (2014). 25047834 PMC4187717

[b19] Li J. F. . Upregulation of microRNA-146a by hepatitis B virus X protein contributes to hepatitis development by downregulating complement factor H. Mbio 6, ARTN e02459-14 10.1128/mBio.02459-14 (2015). PMC4453536 25805734

[b20] Thornburg N. J., Hayward S. L. & Crowe J. E. Respiratory syncytial virus regulates human microRNAs by using mechanisms involving beta interferon and NF-kappa B. Mbio 3, ARTN e00220-12 10.1128/mBio.00220-12 (2012). PMC3529541 23249809

[b21] Zhang B. C., Zhang J. & Sun L. In-depth profiling and analysis of host and viral microRNAs in Japanese flounder (*Paralichthys olivaceus*) infected with megalocytivirus reveal involvement of microRNAs in host-virus interaction in teleost fish. Bmc Genomics 15, Artn 878 10.1186/1471-2164-15-878 (2014). PMC4200114 25297525

[b22] Zhang B. C., Zhang J., Xiao Z. Z. & Sun L. Rock bream (*Oplegnathus fasciatus*) viperin is a virus-responsive protein that modulates innate immunity and promotes resistance against megalocytivirus infection. Dev Comp Immunol 45, 35–42, 10.1016/j.dci.2014.02.001 (2014). 24525178

[b23] Zhu L. Y., Nie L., Zhu G., Xiang L. X. & Shao J. Z. Advances in research of fish immune-relevant genes: A comparative overview of innate and adaptive immunity in teleosts. Dev Comp Immunol 39, 39–62, 10.1016/j.dci.2012.04.001 (2013). 22504163

[b24] Zhang Y. B. & Gui J. F. Molecular regulation of interferon antiviral response in fish. Dev Comp Immunol 38, 193–202, 10.1016/j.dci.2012.06.003 (2012). 22721905

[b25] Song Y. . Early stress responses in Atlantic salmon (*Salmo salar*) exposed to environmentally relevant concentrations of uranium. Aquat Toxicol 112, 62–71, 10.1016/j.aquatox.2012.01.019 (2012). 22366426

[b26] Ohtsuka T. . ASC is a Bax adaptor and regulates the p53-Bax mitochondrial apoptosis pathway. Nat Cell Biol 6, 121−+, 10.1038/ncb1087 (2004). 14730312

[b27] Cayrol C., Knibiehler M. & Ducommun B. p21 binding to PCNA causes G1 and G2 cell cycle arrest in p53-deficient cells. Oncogene 16, 311–320, 10.1038/sj.onc.1201543 (1998). 9467956

[b28] Skalsky R. L. & Cullen B. R. Viruses, microRNAs, and host interactions. Annu Rev Microbiol 64, 123–141, 10.1146/annurev.micro.112408.134243 (2010). 20477536 PMC3621958

[b29] Chen H., Zhang L. R., Yu K. F. & Wang A. M. Pathogenesis of soybean mosaic virus in soybean carrying Rsv1 gene is associated with miRNA and siRNA pathways, and breakdown of AGO1 homeostasis. Virology 476, 395–404, 10.1016/j.virol.2014.12.034 (2015). 25591174

[b30] Cazalla D., Yario T. & Steitz J. Down-regulation of a host microRNA by a herpesvirus saimiri noncoding RNA. Science 328, 1563–1566, 10.1126/science.1187197 (2010). 20558719 PMC3075239

[b31] Lanford R. E. . Therapeutic silencing of microRNA-122 in primates with chronic hepatitis C virus infection. Science 327, 198–201, 10.1126/science.1178178 (2010). 19965718 PMC3436126

[b32] Hussain M. & Asgari S. Functional analysis of a cellular microRNA in insect host-ascovirus interaction. J Virol 84, 612–620, 10.1128/JVI.01794-09 (2010). 19846520 PMC2798455

[b33] O’Connor C. M., Vanicek J. & Murphy E. A. Host microRNA regulation of human cytomegalovirus immediate early protein translation promotes viral latency. J Virol 88, 5524–5532, 10.1128/Jvi.00481-14 (2014). 24599990 PMC4019081

[b34] Murphy E., Vanicek J., Robins H., Shenk T. & Levine A. J. Suppression of immediate-early viral gene expression by herpesvirus-coded microRNAs: implications for latency. P Natl Acad Sci USA 105, 5453–5458, 10.1073/pnas.0711910105 (2008). PMC2291141 18378902

[b35] Broekema N. M. & Imperiale M. J. miRNA regulation of BK polyomavirus replication during early infection. P Natl Acad Sci USA 110, 8200–8205, 10.1073/pnas.1301907110 (2013). PMC3657827 23630296

[b36] Schyth B. D. . Two virus-induced microRNAs known only from teleost fishes are orthologues of micrornas involved in cell cycle control in humans. Plos One 10, ARTN e0132434 10.1371/journal.pone.0132434 (2015). PMC4514678 26207374

[b37] Paludan S. R., Bowie A. G., Horan K. A. & Fitzgerald K. A. Recognition of herpesviruses by the innate immune system. Nat Rev Immunol 11, 143–154, 10.1038/nri2937 (2011). 21267015 PMC3686362

[b38] Garcia-Sastre A. & Biron C. A. Type 1 interferons and the virus-host relationship: a lesson in detente. Science 312, 879–882, 10.1126/science.1125676 (2006). 16690858

[b39] Rasmussen S. B. . Type I interferon production during herpes simplex virus infection is controlled by cell-type-specific viral recognition through toll-like receptor 9, the mitochondrial antiviral signaling protein pathway, and novel recognition systems. J Virol 81, 13315–13324, 10.1128/Jvi.01167-07 (2007). 17913820 PMC2168887

[b40] Forster S. C., Tate M. D. & Hertzog P. J. MicroRNA as type I interferon-regulated transcripts and modulators of the innate immune response. Front Immunol 6, 1–9, Artn 334 10.3389/Fimmu.2015.00334 (2015). 26217335 PMC4495342

[b41] Zhou Z. J. & Sun L. CsCTL1, a teleost C-type lectin that promotes antibacterial and antiviral immune defense in a manner that depends on the conserved EPN motif. Dev Comp Immunol 50, 69–77, 10.1016/j.dci.2015.01.007 (2015). 25636784

[b42] Xu C. Z. . Downregulation of microRNA miR-526a by enterovirus inhibits RIG-I-dependent innate immune response. J Virol 88, 11356–11368, 10.1128/Jvi.01400-14 (2014). 25056901 PMC4178780

[b43] Liang D. G. . A human herpesvirus miRNA attenuates interferon signaling and contributes to maintenance of viral latency by targeting IKK epsilon. Cell Res 21, 793–806, 10.1038/cr.2011.5 (2011). 21221132 PMC3325102

[b44] Hou J. . MicroRNA-146a feedback inhibits RIG-I-dependent type I IFN production in macrophages by targeting TRAF6, IRAK1, and IRAK2. J Immunol 183, 2150–2158, 10.4049/jimmunol.0900707 (2009). 19596990

[b45] Zhou H. B. . miR-155 and its star-form partner miR-155* cooperatively regulate type I interferon production by human plasmacytoid dendritic cells. Blood 116, 5885–5894, 10.1182/blood-2010-04-280156 (2010). 20852130

[b46] Peng W. Z., Ma R., Wang F., Yu J. & Liu Z. B. Role of miR-191/425 cluster in tumorigenesis and diagnosis of gastric cancer. Int J Mol Sci 15, 4031–4048, 10.3390/ijms15034031 (2014). 24603541 PMC3975382

[b47] Wang S. F. . Loss of microRNA 122 expression in patients with hepatitis B enhances hepatitis B virus replication through cyclin G1-modulated P53 activity. Hepatology 55, 730–741, 10.1002/hep.24809 (2012). 22105316

[b48] Gottwein E. & Cullen B. R. A Human Herpesvirus microRNA inhibits p21 expression and attenuates p21-mediated cell cycle arrest. J Virol 84, 5229–5237, 10.1128/Jvi.00202-10 (2010). 20219912 PMC2863803

[b49] Zhang M., Xiao Z. Z., Hu Y. H. & Sun L. Characterization of a megalocytivirus from cultured rock bream, *Oplegnathus fasciatus* (Temminck & Schlege), in China. Aquac Res 43, 556–564, 10.1111/j.1365-2109.2011.02861.x (2012).

[b50] Zhang J., Hu Y. H., Sun B. G., Xiao Z. Z. & Sun L. Selection of normalization factors for quantitative real time RT-PCR studies in Japanese flounder (*Paralichthys olivaceus*) and turbot (*Scophthalmus maximus*) under conditions of viral infection. Vet Immunol Immunop 152, 303–316, 10.1016/j.vetimm.2012.12.018 (2013). 23332581

[b51] Zhang B. C. & Sun L. Tongue sole (*Cynoglossus semilaevis*) prothymosin alpha: cytokine-like activities associated with the intact protein and the C-terminal region that lead to antiviral immunity via Myd88-dependent and -independent pathways respectively. Dev Comp Immunol 53, 96–104, 10.1016/j.dci.2015.07.004 (2015). 26162512

[b52] Tong S. L., Li H. & Miao H. Z. The establishment and partial characterization of a continuous fish cell line FG-9307 from the gill of flounder Paralichthys olivaceus. Aquaculture 156, 327–333, 10.1016/S0044-8486(97)00070-7 (1997).

[b53] Zhou Z. X., Zhang B. C. & Sun L. Poly(I:C) induces antiviral immune responses in Japanese flounder (*Paralichthys olivaceus*) that require TLR3 and MDA5 and is negatively regulated by Myd88. Plos One 9, ARTN e112918 10.1371/journal.pone.0112918 (2014). PMC4231074 25393122

[b54] Ohtani M. . Transcriptional regulation of type I interferon gene expression by interferon regulatory factor-3 in Japanese flounder. Paralichthys olivaceus. Dev Comp Immunol 36, 697–706, 10.1016/j.dci.2011.10.008 (2012). 22067740

[b55] Zhou B. S. . MicroRNA-503 targets FGF2 and VEGFA and inhibits tumor angiogenesis and growth. Cancer Lett 333, 159–169, 10.1016/j.canlet.2013.01.028 (2013). 23352645

