# Supplementary material for: pol-miR-731, a teleost miRNA upregulated by megalocytivirus, negatively regulates virus-induced type I interferon response, apoptosis, and cell cycle arrest
Source: Sci Rep. 2016 Jun 17;6:28354. doi: 10.1038/srep28354 (PMC4911600; doi:10.1038/srep28354)

Supplementary Information

pol-miR-731, a teleost miRNA hijacked by megalocytivirus, negatively regulates virus-induced type I interferon response, apoptosis, and cell cycle arrest

Bao-cun Zhang[1,2], Ze-jun Zhou[1,2,3], Li Sun[1,2]

Table S1. Flounder miRNAs significantly altered in expression at 1 d to 6 d post-viral infection.

Time post infection (day)	Upregulated miRNA	Downregulated miRNA
1	pol-miR-456 pol-miR-155	pol-miR-22-3p
2	pol-miR-204	pol-let-7e<br>pol-miR-140-3p
3	pol-miR-204 pol-miR-155<br>pol-miR-221-5p pol-miR-183<br>pol-let-7f	pol-miR-126b-3p<br>pol-let-7e<br>pol-miR-93<br>pol-miR-30e-3p
4	pol-miR-183 pol-miR-731<br>pol-let-7f pol-miR-456<br>pol-miR-155 pol-miR-204	pol-miR-222-p5<br>pol-let-7e<br>pol-miR-30e-3p
5	pol-miR-183 pol-miR-204<br>pol-miR-221-5p<br>pol-miR-221-3p<br>pol-miR-731 pol-miR-155	pol-miR-5p-96_67419
6	pol-miR-183 pol-miR-221-5p<br>pol-miR-204 pol-miR-155<br>pol-let-7f pol-miR-456	pol-miR-3p-49_126683

Table S2. Sequences of synthesized small RNAs used in the study.

Name	Sequence (5’-3’)
pol-miR-731mimic	AAUGACACGUUUUCUCCCGGAU
pol-miR-731 mimic-Mut	AUACUGUGGUUUUCUCCCGGAU
pol-miR-731agomir	AAUGACACGUUUUCUCCCGGAU
pol-miR-731antagomir	UUCCGGGUGUUUUCGUGUCUUU
mimic control	UCACAACCUCCUAGAAAGAGUAGA
agomir negative control	UUUGUACUACACAAAAGUACUG
antagomir negative control	UUUGUACUACACAAAAGUACUG

Table S3. Sequences of the primers used in the study.

Primer	Sequence (5'-3')
F1	GATATCGCCACCATGCAAAGCCTTCCAAAG (EcoRV)
R1	GATATCATGCAGGAAGGTCGGG (EcoRV)
F2	CCCGGGGCCACCATGGAAGAGCAAGGTTTG (SmaI)
R2	CCCGGGGTCACTGTCGCTCTGCTC (SmaI)
F3	ACTAGTATCTCAAACTTCTTCAGCACTG (Spe I)
R3	AAGCTTTCAAATACAAAATGACCAGCA (HindIII)
F4	ACTAGTGAAGCTCGTTGCCTTGAT(Spe I)
R4	AAGCTTCCAATTAAAATCTGATTAAAATGA (HindIII)
F5	ACGCGTGACACACGTGTGAAAAACAC (MluI)
R5	AGATCTTGTTGGAGCTGACCTGC (BglII)

Note: Underlined nucleotides are restriction sites of the enzymes indicated in the brackets at the ends.

Figure S1. Detection of plasmids (A) and expression of plasmid-derived immune genes (B) in flounder. (A) Flounder were administered with pCN3 (lane 6, 7, and 8), pCNIRF7 (lane 4), pCNp53 (lane 5), and PBS (lanes 2, 3, and 9). At 4 d post-plasmid administration, DNA was extracted from spleen and used for PCR with primers specific to pCNIRF7 (lanes 2, 4, and 6), pCNp53 (lanes 3, 5, and 7), and pCN3 (lanes 8 and 9). (B) Flounder were administered with pCNIRF7 (lane 4), pCNp53 (lane 5), pCN3 (lanes 2 and 3), and PBS (lanes 6 and 7). At 4 d after plasmid administration, RNA was extracted from spleen and used for RT-PCR with primers specific to plasmid-derived pCNIRF7 (lanes 2, 4, and 6) and pCNp53 (lanes 3, 5, and 7), or, as an internal control, with primers specific to EF1A (lower panel). Lane 1 of both panels, DNA molecular weight markers.

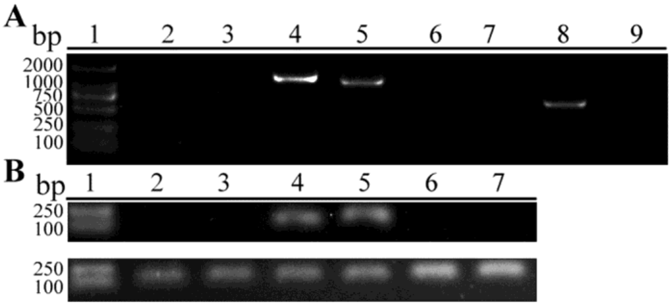

Figure S2. Effect of pol-miR-731 on the expression of type I interferon (IFN) and IFN-stimulated genes under the condition of IRF7 overexpression. Flounder infected with megalocytivirus RBIV-C1 were administered with pol-miR-731 agomir plus pCNIRF7 or the control plasmid pCN3, and the mRNA levels of IFN (A, upper panel) and IFN-stimulated genes (B) were determined by qRT-PCR. The protein level of IFN was determined by Western blot (A, lower panel). All experiments were perfromed three times, and values are shown as means ± SEM. **P* < 0.05, ***P* < 0.01.

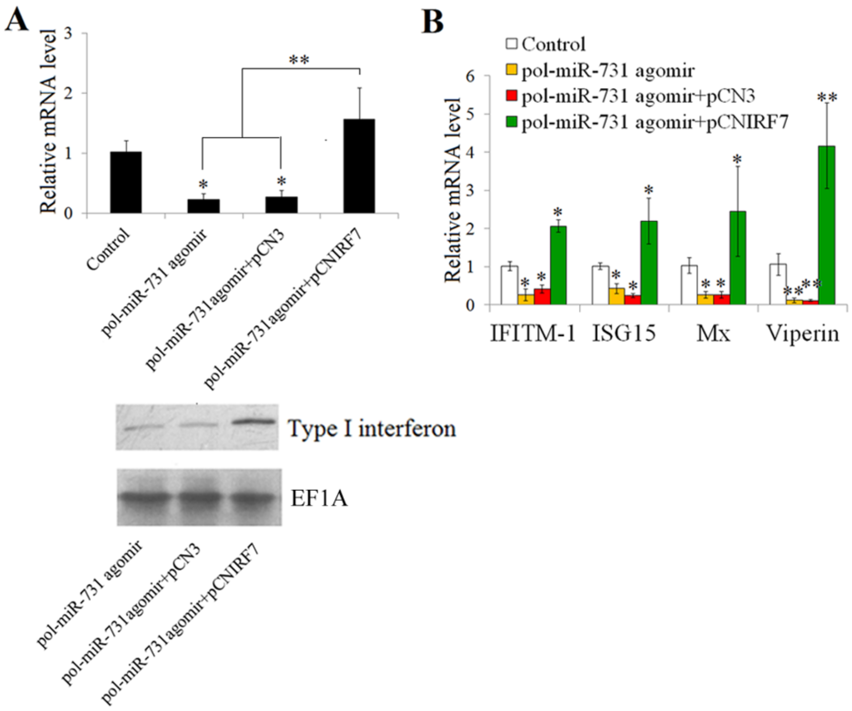

Figure S3. Effect of pol-miR-731 on apoptosis under the condition of p53 overexpression. Flounder were administered with pol-miR-731 agomir plus pCNp53 or the control plasmid pCN3, and apoptosis of splenocytes was assayed at 4 days post-infection (A). The assay was performed three times, and significant differences in values are indicated by different letters. The results of one representative experiment are shown in panel (B).

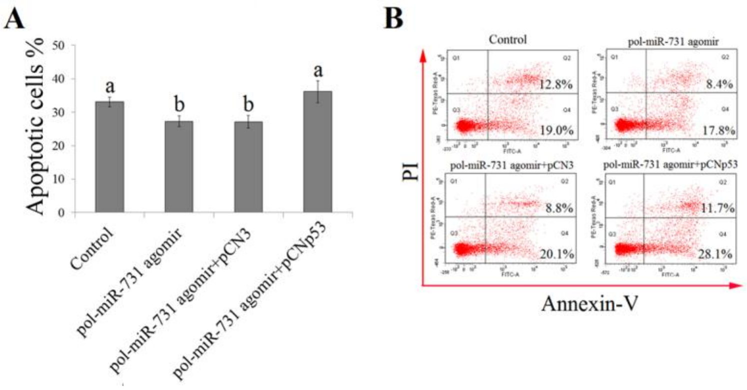

Figure S4. Effect of pol-miR-731 on cell cycle arrest under the condition of p53 overexpression. Flounder were administered with pol-miR-731 agomir plus pCNp53 or pCN3, and cell cycle arrest of splenocytes was assayed at 4 days post-infection (A). The assay was performed three times, and values are shown as means ± SEM. *P < 0.05. The results of one representative experiment are shown in panel (B).

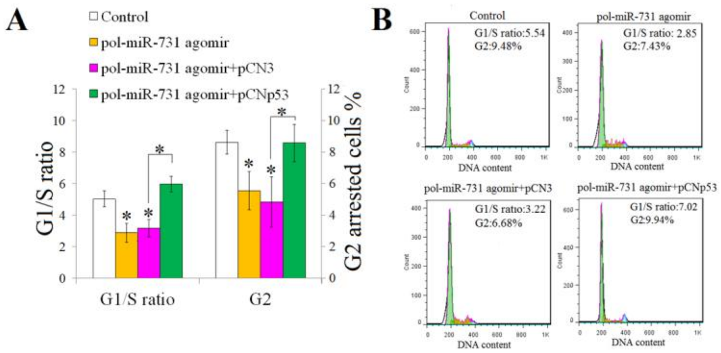

Supplement: Supplementary Information [file srep28354-s1.pdf]
